# Feasibility of measuring lumbar, postural, and biomechanical outcomes before and after a Rolfing® Structural Integration 10-series delivered by trainees: a single-arm pilot study

**DOI:** 10.1186/s40814-026-01954-z

**Published:** 2026-11-10

**Authors:** L. Overmann, C. Verleger, R. Schleip, A. Brandl, C. Veeck, P. Weber, R. Löw, K. Bartsch

**Affiliations:** 1 Association for Fascia Research, Munich, 80799 Germany; 2 Ida P Rolf Research Foundation, Boulder, CO USA; 3 Department of Psychology, FernUniversität Hagen, Hagen, Germany; 4 Associated Professorship of Conservative and Rehabilitative Orthopedics, TUM School of Medicine and Health, Technical University of Munich, Munich, 80809 Germany; 5 Deutsche Gesellschaft Für Myofascial Release E.V., Munich, Germany; 6 Department of Physical Activity in Public Health, Institute of Movement and Neurosciences, German Sport University Cologne, Cologne, Germany; 7 Department of Physiotherapy, Medical Cooperative Society, PANOVIA, Hürth, Germany; 8 Orthopaedic Practice Burgdorf, Weserstraße 6, Burgdorf, 31303 Germany

**Keywords:** Structural integration, Pilot study, Feasibility study, DIERS formetric 4D, Posture, Fascia, Manual therapy, Trainee-delivered intervention

## Abstract

**Background:**

Rolfing® Structural Integration (RSI) is a manual and movement-education approach, traditionally delivered as a 10-session series, that aims to support postural organization, movement efficiency, and body awareness. Although the evidence base has expanded, prospective studies using standardized objective outcomes remain scarce. Before an adequately powered controlled trial can be justified, researchers should establish the feasibility of delivering the 10-series in an educational training setting and collecting objective postural, sensory, physiological, and biomechanical outcomes.

**Methods:**

This single-arm, non-randomized pilot and feasibility study used pre–post measurements around a Rolfing® Structural Integration 10-series delivered in an educational training setting in Munich, Germany. Rolfers-in-training delivered the intervention under senior supervision. Feasibility outcomes included recruitment, eligibility, retention, intervention completion, post-treatment measurement completion, data availability, and adverse-event data capture. Exploratory postural outcomes included individual DIERS formetric 4D rasterstereography parameters and an unvalidated exploratory z-normalized postural imbalance index. Secondary/exploratory outcomes included DIERS parameters, pressure pain threshold, perceived pain assessed using a visual analog scale, subject expectation, skin temperature as a potential confounding variable, and MyotonPro and IndentoPro measures.

**Results:**

The final analysis included *n* = 10 participants with complete pre–post instrumental data. Recruitment, retention, post-treatment assessment completion, DIERS measurement standardization, provider background documentation, and completeness of patient-reported outcome data were identified as key feasibility domains. Exploratory instrumental outcomes, including the unvalidated z-normalized postural imbalance index, could be summarized descriptively, but the small, uncontrolled sample was not designed to evaluate the intervention’s efficacy.

**Conclusion:**

The RSI 10-series and the objective pre- and post-measurement protocol were feasible in principle within an educational training setting. Limitations included a small final sample size, attrition, incomplete provider background documentation, and challenges with DIERS standardization during on-site training. Future studies should improve retention, optimize DIERS procedures, document the training and supervision context, and ensure complete capture of patient-reported outcome data.

**Supplementary Information:**

The online version contains supplementary material available at https://doi.org/10.1186/s40814-026-01954-z.

## Key messages regarding feasibility

- What uncertainties existed regarding the feasibility?
  It was unclear whether a trainee-delivered Rolfing® Structural Integration 10-series in an educational setting could be combined with repeated objective measurements of posture, biomechanics, sensory function, physiology, and patient-reported outcomes. Key uncertainties concerned recruitment, retention, post-treatment assessment completion, DIERS measurement standardization, and adverse-event data capture.
- What are the key feasibility findings?
  The intervention and objective measurement protocol were, in principle, feasible for participants who remained in the study. Of 19 potential participants, 15 completed the baseline assessment, and 10 completed both baseline and post-intervention assessments. Key feasibility challenges included attrition, scheduling within the educational training setting, DIERS measurement standardization, incomplete provider background documentation, loss of quantitative VAS data, and limited interpretability of two follow-up activity-restriction questionnaire entries.
- What are the implications of the feasibility findings for the design of the main study?
  A future adequately powered trial should strengthen recruitment and retention procedures, define post-treatment assessment windows, standardize DIERS setup and landmarking, document trainee background and supervision context, and integrate complete capture of patient-reported outcomes and adverse events. Future studies should prioritize feasibility procedures, measurement standardization, and established individual postural and biomechanical variables rather than relying on an unvalidated composite score as a primary outcome.

## Introduction

Optimal postural alignment and efficient movement are key components of musculoskeletal health and human performance. Both depend not only on neuromuscular control and joint mobility but also on the mechanical and sensory properties of connective tissues. In this context, attention has increasingly focused on the body’s fascial network, a body-wide connective tissue system involved in force transmission, proprioception, movement coordination, and adaptation to mechanical loading [1–4]. Because fascia contributes to mechanical load transfer and sensorimotor regulation, myofascial tissue properties are relevant to consider when developing measurement protocols for manual and movement-based therapies such as RSI. However, the present pilot study was not designed to test whether changes in fascial properties mediate potential effects of RSI.

Rolfing® Structural Integration, hereafter referred to as RSI, is one such approach. RSI is traditionally delivered as a 10-session series and combines manual work on myofascial tissues with movement and perception-oriented education. The method has been described as aiming to align and balance the body in relation to gravity, thereby supporting spatial control, movement capacity, and whole-body functional organization [5–7]. RSI was selected for the present feasibility study because its 10-session series provides a recognizable and teachable intervention framework that combines manual myofascial work with movement and perception-oriented education, making it suitable for studying the feasibility of repeated objective measurements around a complete intervention sequence in an educational training setting. Although RSI is used in both clinical and non-clinical contexts, including by individuals seeking improvements in posture, movement quality, body awareness, or musculoskeletal well-being, prospective feasibility work is needed before objective postural and biomechanical parameters can be evaluated in adequately powered controlled studies.

The empirical evidence base for RSI has expanded in recent years but remains heterogeneous. Earlier literature consisted largely of case reports, small studies, and clinically oriented reports, highlighting the need for more rigorous prospective research [7–9]. More recent studies have examined RSI or RSI-related interventions in different populations. In a retrospective cohort study, Brandl et al. [10] analyzed data from 383 clients who completed 10 RSI sessions and reported pre–post changes in active range of motion of the shoulder and hip. Randomized studies have explored RSI-related interventions in specific populations, including pelvic floor muscle facilitation in healthy women and gross motor function and gait in young children with spastic cerebral palsy [11, 12]. Overall, the available evidence remains heterogeneous across study populations, intervention contexts, comparator conditions, and outcome domains. The interpretation of this literature is further limited by small sample sizes in some studies, retrospective designs, limited use of control conditions, population heterogeneity, variation in RSI-related protocols, and potential risk of bias.

The lower back is a particularly relevant region for investigating biomechanical and postural outcomes in RSI research. The thoracolumbar fascia has been described as relevant for upright posture, movement in gravity, and spinal stabilization, and has received increasing attention in anatomical, biomechanical, and clinical research [3, 13–15]. Tissue stiffness is a key biomechanical property of fascia and can be understood as the resistance of tissue to external deformation; it may be influenced by factors such as hydration, cross-linking, molecular organization, disease, aging, and mechanical loading [4, 16, 17]. In the present feasibility study, lower-back tissue stiffness was assessed as an exploratory mechanical tissue-property measure, not as a test of pain mechanisms or pathology. These considerations support the inclusion of lower-back tissue-property measurements in feasibility studies examining RSI. The lower back was selected not as a proxy for whole-body treatment effects, but as an anatomically relevant, practically accessible, and repeatable measurement region related to the thoracolumbar fascia and suitable for standardizing local tissue-property assessments within a multimodal pilot protocol. A characteristic of the existing RSI literature is that many studies have focused on range-of-motion measures, subjective reports, or population-specific clinical outcomes. The measurement battery was therefore selected to examine the feasibility of collecting data across several predefined measurement domains rather than to test separate clinical hypotheses. DIERS rasterstereography was used to characterize postural alignment and spinal surface parameters; pressure algometry was used to assess pressure pain sensitivity; MyotonPro and IndentoPro measures were used to assess exploratory mechanical tissue properties; and local skin temperature was recorded as contextual information and a potential confounder for mechanical tissue-property measurements. DIERS formetric 4D rasterstereography has been used in numerous studies and has shown generally good reliability for many spine-shape parameters, although reliability varies between individual parameters [18]. Reference values for videorasterstereographic spinal posture in healthy adults have also recently been reported, supporting the relevance of standardized postural assessment in research and clinical contexts [19]. However, no previous prospective RSI study has established whether a complete RSI 10-series delivered in an educational training setting can be combined with repeated objective measurements using DIERS rasterstereography, pressure algometry, myotonometry, indentometry, skin temperature assessment, and patient-reported outcome and adverse-event capture. This leaves an important feasibility gap regarding recruitment, retention, measurement standardization, data completeness, participant burden, and the interpretability of exploratory objective outcomes in this setting.

Therefore, this single-arm pre–post pilot and feasibility study was conducted to evaluate whether a supervised RSI 10-series delivered in an educational training setting can be combined with a multimodal pre–post assessment protocol.

The primary feasibility objectives were to assess recruitment, eligibility, retention, completion of the 10-session intervention, completion of post-treatment assessment, availability of analyzable pre–post instrumental data, adverse-event data capture, and procedural challenges related to measurement standardization and participant burden. Secondary patient-centered and exploratory objectives were to assess whether postural alignment, lower-back mechanical tissue parameters, pressure pain threshold, local skin temperature, and patient-reported lower-back outcomes could be collected, summarized, and interpreted descriptively within this study setting. These outcomes were included to inform outcome selection, variability estimates, and measurement procedures for a future adequately powered trial, not to provide confirmatory evidence of clinical effectiveness.

## Methods

### Study design and setting

This single-arm, non-randomized pilot and feasibility study used repeated pre- and post–measurements to evaluate the feasibility of collecting objective biomechanical, postural, sensory, and patient-reported data before and after a complete RSI 10-series. The intervention was conducted over 6 weeks, corresponding to the planned duration of the 10-series within the Intensive Basic RSI Training at the European Rolfing® Association e.V. (ERA) Training Center in Munich, Germany. This setting was selected because the complete RSI 10-series was delivered to external training models within a defined educational program, enabling evaluation of the feasibility of repeated objective measurements across a complete intervention sequence.

Ethical approval was granted by the Ethics Committee of the German Sport University Cologne, Germany (application number 051/2021), with the approval/no-objection letter issued on 31 March 2021. The research was conducted in accordance with the principles of the Declaration of Helsinki. Prospective registration was completed with the German Clinical Trials Register on 22 June 2021 (DRKS00024746), before participant recruitment. Written informed consent was obtained from all participants before inclusion.

Reporting followed the guidance for non-randomized pilot and feasibility studies by Lancaster and Thabane [20], which recommends adapting the CONSORT extension for pilot and feasibility trials to non-randomized designs where applicable.

### Participants

Participants were recruited from external models scheduled to receive an RSI 10-series during the Intensive Basic RSI Training at the ERA Training Center in Munich, Germany. In this training context, external models receive the 10-series as part of the practical education of Rolfers-in-training. Participation in the research study was voluntary and independent of participation as a training model.

Eligible participants were external models scheduled for the complete RSI 10-series at the ERA Training Center, aged 18 to 65 years, and able to complete German-language study procedures. According to the general ERA model criteria, participants were additionally expected to have no chronic, acute, inflammatory, or dermatological disease, no current psychotherapeutic treatment, and no previous RSI 10-series within the preceding 3–5 years.

Exclusion criteria for the research study were acute musculoskeletal injury; impairments of hearing, speech, vision, or mobility that would interfere with participation; pregnancy; implanted medical devices such as a cardiac pacemaker; osteoporosis; blood-clotting disorders; eczema; skin infection; venous thrombosis; burns; acute trauma including unhealed fractures or open wounds; psoriasis; poorly controlled psychiatric illness; fibromyalgia; implanted rods, screws, or plates; previous discectomy; medication with anticoagulants or immunosuppressants; chronic steroid medication; daily narcotic analgesic use; alcohol or substance abuse; chronic conditions such as type I diabetes, Crohn’s disease, lupus, irritable bowel syndrome, or autoimmune disease; BMI ≥ 40; and any other relevant medical condition that could impair participation in study procedures.

### Intervention

The intervention consisted of the RSI 10-series, a structured manual-and-sensorimotor education approach traditionally delivered over 10 sequential sessions. The RSI 10-series follows a predefined session sequence with a standardized progression from superficial “sleeve” work, through deeper “core” sessions, to final integration sessions [6, 7].

Sessions 1–3 focused on superficial “sleeve” work, referring to work with more superficial myofascial layers and regions related to breathing, support through the feet, and organization of the lateral body. Sessions 4–7 addressed deeper “core” work, referring to work with deeper myofascial and coordinative structures related to pelvic, spinal, and head–neck organization. Sessions 8–10 focused on “integration,” referring to linking upper- and lower-body organization with whole-body movement, perception, and coordination. Although the sequence and general therapeutic goals of the 10-series were standardized, specific manual contacts, pressure, pacing, tissue targets, and movement or perception cues were adapted to each participant’s presentation. Thus, standardization referred to the overall 10-session framework, session sequence, and general session goals, whereas the specific manual strategies and movement or perception cues were individualized. Participants received the complete 10-series over six weeks during the Intensive Basic Rolfing® Training at the ERA Training Center in Munich, Germany. Consistent with typical Rolfing® practice, individual sessions lasted approximately 60–90 min, although the exact duration could vary according to the session goals and the participant’s presentation. Treatments were delivered by Rolfers-in-training in the final part of their education and supervised by experienced senior teachers. Individual trainees’ prior clinical, manual therapy, or bodywork experience was not systematically documented in the study dataset and therefore could not be summarized quantitatively. Participant-to-trainee assignment and participant-level variation in manual contact, pressure, pacing, and tissue targets were not quantified as separate intervention-delivery or intervention-dose variables in the present study. Senior supervision was used to support delivery within the intended educational framework; however, no formal treatment-fidelity checklist, independent fidelity rating, or structured session-level documentation of protocol adherence was collected for the present analysis. Future studies should document key elements of treatment delivery, trainee background, participant-to-trainee assignment, session duration, and fidelity procedures prospectively to better characterize intervention delivery while preserving the individualized nature of RSI.

### Primary feasibility objectives

The primary feasibility objectives were recruitment, eligibility, retention through the 10-session intervention, completion of post-treatment assessment, availability of analyzable pre-post instrumental data, adverse-event data capture, and procedural challenges related to measurement standardization and participant burden. These outcomes were assessed using participant-flow documentation, completion of baseline and post-intervention assessments, availability of analyzable instrumental datasets, questionnaire completion, and narrative documentation of procedural or organizational challenges during the study.

### Secondary patient-centered and exploratory objectives

Secondary patient-centered and exploratory objectives included postural alignment, pressure pain threshold, local skin temperature, lower-back mechanical tissue parameters, and patient-reported lower-back symptoms and activity restriction. These outcomes were measured before and after the RSI 10-series to assess data collection procedures, estimate variability, evaluate interpretability, and inform outcome selection for a future adequately powered trial. They were not intended to test the efficacy of the intervention.

### Postural alignment

Postural alignment was assessed at baseline and follow-up using the DIERS formetric 4D system. DIERS formetric 4D is an optical rasterstereography system that projects parallel light lines onto the posterior trunk, records the projected light pattern with an imaging unit, and generates a three-dimensional surface model of the back. Rasterstereography provides a radiation-free method for assessing spinal and postural surface parameters. Previous reliability research in adults without postural deformities reported generally good to excellent reliability for most DIERS formetric 4D spine-shape parameters, although reliability varies by parameter [18].

Baseline and follow-up measurements were performed in a relaxed standing position with the posterior trunk exposed and the back facing the DIERS camera. Participants stood with their arms resting at their sides, and head and gaze were oriented forward during the measurement. Participants were positioned on the PedoScan platform to support repeatable foot placement and standing position across baseline and follow-up assessments. Room and lighting conditions were controlled as far as possible, and direct or disturbing light sources were avoided during DIERS recording. Reflective markers were not required for standard DIERS formetric 4D assessment because the system automatically detects anatomical landmarks. In the present on-site setting, however, practical challenges related to landmark identification, marker placement, and marker adhesion occurred during data collection and processing. The exact device-to-participant distance was not documented as a separate study variable. DIERS measurements were performed by two members of the study team involved in DIERS data collection and processing, one of whom had several years of practical experience using the DIERS system.

The recorded DIERS parameters included pelvic inclination in the sagittal plane, lordotic angle, kyphotic angle, trunk inclination, pelvic tilt in the coronal plane, and trunk imbalance. These parameters were selected as exploratory descriptors of sagittal and coronal postural alignment and spine-shape characteristics, rather than as hypothesis-specific clinical endpoints. Their inclusion was intended to evaluate the feasibility and interpretability of objective postural assessment within a multimodal pilot protocol.

An exploratory z-normalized postural imbalance index was calculated from three predefined DIERS parameters: trunk inclination, trunk imbalance, and pelvic inclination. This index was not a validated clinical endpoint and was used only as a descriptive exploratory measure. The individual DIERS parameters were prioritized for interpretation because they are more transparent than the composite index and do not imply validation of the equally weighted composite score.

In some participants, more than one DIERS recording was obtained at a given measurement time point when the initial recording was considered insufficient for further processing. The final descriptive analysis was based on selected baseline and follow-up recordings judged comparable, particularly for landmark identification, marker placement, marker adhesion, and device-height settings. This selection was not blinded to measurement time point. The number of repeated recordings per participant and time point was not predefined as a separate study variable, and no predefined aggregation procedure (mean or median) was used for repeated recordings. Within-session repeatability, intrarater reliability, and interrater reliability of DIERS measurements were not assessed in the current sample. These issues were treated as measurement-standardization limitations and feasibility findings for future protocol development.

### Standardization of measurement sites for local assessments

Local tissue assessments were performed at two predefined anatomical regions: the lower lumbar region and the forearm. These regions were selected to provide one intervention-relevant trunk measurement site and one peripheral comparison site. To ensure local skin temperature, MyotonPro, IndentoPro, and pressure pain threshold measurements were taken at consistent locations within each participant, we marked the measurement sites before the local tissue assessments.

For the lower-back assessment, participants were positioned prone on a treatment table, with the feet extending beyond the end of the table. The lumbar measurement point was marked on the right side, 2.5 cm lateral to the L3 spinous process. For the forearm pressure pain threshold assessment, participants were positioned seated, with the forearm and dorsal side of the hand resting on the treatment table. The measurement point was marked on the palmar side of the left forearm, 15 cm proximal to the wrist and centrally between the medial and lateral borders of the forearm.

### Local skin temperature

Local skin temperature at the lower-back measurement site was recorded before mechanical tissue-property assessments as a contextual physiological variable and a potential confounder for tissue-property measurements. Skin temperature was recorded in degrees Celsius using a multifunctional thermometer, such as the FT-90 device by Beurer GmbH. Infrared thermometry is a non-invasive method for assessing skin temperature, and previous research supports the acceptable reliability of repeated infrared skin-temperature measurements under standardized conditions [21].

### Myotonometric assessment of tissue mechanical properties

Mechanical tissue parameters were assessed using the MyotonPro device. Myotonometry is a non-invasive method that applies a brief mechanical impulse to the tissue and records the resulting damped oscillation. The device provides parameters such as dynamic stiffness and oscillation frequency. Dynamic stiffness is a device-specific parameter, reported in N/m, that represents an oscillation-based estimate of local tissue resistance to a brief mechanical impulse rather than a direct measure of intrinsic biomechanical stiffness. Oscillation frequency is reported in Hz. Reliability and construct validity studies support the use of MyotonPro-derived parameters to assess the mechanical properties of musculoskeletal tissues under standardized testing conditions, with high-to-very-high intra-rater, inter-rater, and inter-session reliability reported in previous research [22, 23].

MyotonPro measurements were performed at the predefined lower-back measurement site. Five repeated measurements were obtained. Dynamic stiffness, oscillation frequency, and the coefficient of variation for both parameters were recorded. Invalid measurements were repeated.

### Indentometry-based tissue stiffness

Indentometry-based tissue stiffness was assessed using the IndentoPro device. Indentometry quantifies the mechanical resistance of soft tissue by applying a controlled indentation. A reliability study of the IndentoPro reported moderate inter-rater and good intra-rater reliability for myofascial tissue stiffness measurements, supporting its use as an exploratory tool for assessing myofascial tissue properties [24].

IndentoPro stiffness measurements were performed at the predefined lower-back measurement sites. Stiffness was recorded in N/mm. Three repeated stiffness measurements were performed at each site. The coefficient of variation was recorded, and measurements were repeated if it exceeded 10%. The indentation depth was predefined at 8 mm per the study’s measurement protocol. The participant’s respiratory phase during indentation was not specified as a controlled variable in the study protocol.

### Pressure pain threshold

Pressure pain threshold was assessed using pressure algometry. Pressure pain threshold was defined as the point at which the sensation of pressure first shifted to pain. Pressure algometry is commonly used in musculoskeletal and pain research to quantify mechanical pain sensitivity. Recent systematic review evidence supports its potential reliability in assessing pressure pain threshold in individuals with low back or neck pain, while also emphasizing the importance of standardized testing procedures and consistent measurement protocols [25].

The pressure pain threshold procedure followed standardized verbal instructions adapted from the quantitative sensory testing profile of the German Research Network on Neuropathic Pain [26]. Participants were instructed that the test assessed pain sensitivity rather than pain tolerance. Before testing, the investigator explained the procedure using the following standardized instruction: “This is a test of your sensitivity to pain. I will now press this pressure-measuring device against your muscle and slowly increase the pressure. Please say ‘now’ as soon as the pressure starts to become painful. Remember that this is not a pain tolerance test, but a pain threshold test.” During the test, participants were instructed to say “now” when pressure sensation changed into a burning, stabbing, drilling, pulling, or otherwise painful sensation. Pressure application was stopped immediately after this verbal indication. The measurement was recorded in Newtons.

Pressure pain threshold was assessed at the predefined lower-back and forearm measurement sites. Three repeated measurements were performed at each site, and values were recorded in Newtons.

### Patient-reported outcomes and adverse-event capture

Patient-reported outcomes were collected using German-language study questionnaires at baseline, mid-series, and follow-up. Participants were instructed to complete the questionnaires undisturbed and without influence from others. The questionnaires assessed demographic and eligibility-related information, previous myofascial treatment experience, physical activity, subjective lower-back mobility, current lower-back pain, activity restriction due to lower-back problems, and treatment-related positive or negative experiences. The full questionnaire structure and item content are provided in Supplementary Material S1.

The mid-series questionnaire was administered after the fifth RSI session and included a structured capture of adverse events. Participants indicated whether predefined sensory, autonomic, emotional, movement-related, body-perception-related, breathing-related, or pain-related experiences occurred during or after treatment, documented their duration using predefined categories, and could provide additional free-text descriptions. At follow-up, after completion of the RSI 10-series, patient-reported items on lower-back mobility, pain, and activity restriction were repeated. Adverse-event capture was treated as a feasibility outcome to evaluate whether adverse events could be systematically documented within the study workflow, rather than to estimate their incidence or assess safety.

### Sample size and progression criteria

No formal a priori sample size calculation for hypothesis testing was performed because this was a pilot and feasibility study and was not designed or powered to test intervention efficacy or estimate treatment effects with statistical precision. The sample size was determined pragmatically based on participant availability in the educational training setting and the logistical constraints of delivering a complete RSI 10-series alongside a comprehensive measurement protocol. Pragmatic feasibility progression criteria were defined a priori to evaluate whether a future controlled trial would be practicable. These included recruitment of at least 70% of the target sample, completion of the full 10-session intervention in at least 70% of enrolled participants, and availability of analyzable pre–post instrumental data in at least 60% of enrolled participants. These thresholds were study-specific feasibility criteria rather than empirically validated cut-offs. They were intended to indicate whether recruitment, retention, intervention completion, and availability of instrumental data would be sufficient to justify refining the protocol for a future controlled study. The final sample consisted of *n* = 10 participants who completed the RSI 10-series and attended both baseline and post-intervention assessments. This sample size allowed only a preliminary descriptive assessment of participant flow, retention, intervention completion, data availability, and practical measurement procedures. It did not allow reliable estimation of treatment effects, stable estimates of variability, or confirmatory evaluation of any clinical or biomechanical outcome. Findings were therefore interpreted as preliminary feasibility data and used to inform outcome selection and design considerations for future studies.

### Statistical analysis

All analyses were conducted in line with the pilot and feasibility purpose of the study. Primary feasibility outcomes were analyzed descriptively. Recruitment, eligibility, retention through the 10-session intervention, completion of post-treatment assessment, availability of analyzable pre–post instrumental data, and adverse-event data capture were summarized using absolute numbers and, where appropriate, percentages. Reasons for non-participation, withdrawal, or missing final-analysis data were described narratively.

Secondary patient-centered and exploratory outcomes were summarized descriptively at baseline and post-treatment using means and standard deviations. Pre–post changes were calculated as post-treatment minus baseline values and summarized descriptively. No inferential interpretation was based on confidence intervals or hypothesis tests, because the study was not powered to estimate treatment effects with statistical precision. Because of the small sample size and pilot character of the study, these analyses were interpreted as exploratory and hypothesis-generating rather than confirmatory [27].

The DIERS-derived z-normalized postural imbalance index was treated as a strictly exploratory descriptive postural measure. It was calculated from three predefined DIERS parameters: trunk inclination, trunk imbalance, and pelvic inclination. Each component was z-normalized before aggregation, and the resulting index was calculated separately for baseline and post-treatment assessment. The three components were weighted equally; however, this weighting was not based on prior biomechanical validation. The index was not calculated against external normative values and was not treated as a validated clinical endpoint. It was used only to summarize exploratory postural data descriptively, and no substantive biomechanical or clinical conclusions were drawn from the composite index. The individual DIERS parameters were reported descriptively to provide a more transparent presentation of the postural measurements. No hypothesis testing was performed. The analyses focused on descriptive statistics and mean pre–post changes to describe the direction of observed changes without implying inferential precision. Individual participant trajectories were examined descriptively for the DIERS sum score and selected secondary outcomes to identify heterogeneity in response patterns, because group-level summaries may obscure individual changes in small samples. All other instrumental outcomes, including additional individual DIERS parameters, pressure pain threshold, local skin temperature, MyotonPro-derived dynamic stiffness and oscillation frequency, and IndentoPro-derived stiffness, were treated as exploratory secondary outcomes. Given the pilot character of the study and the large number of exploratory outcomes, these results were interpreted descriptively and considered hypothesis-generating. Statistical analyses were performed using jamovi version 2.3 and R version 4.1. To support reproducibility, R packages were retrieved from an MRAN snapshot dated 1 January 2022.

## Results

### Participant flow and feasibility outcomes

**Fig. 1:**
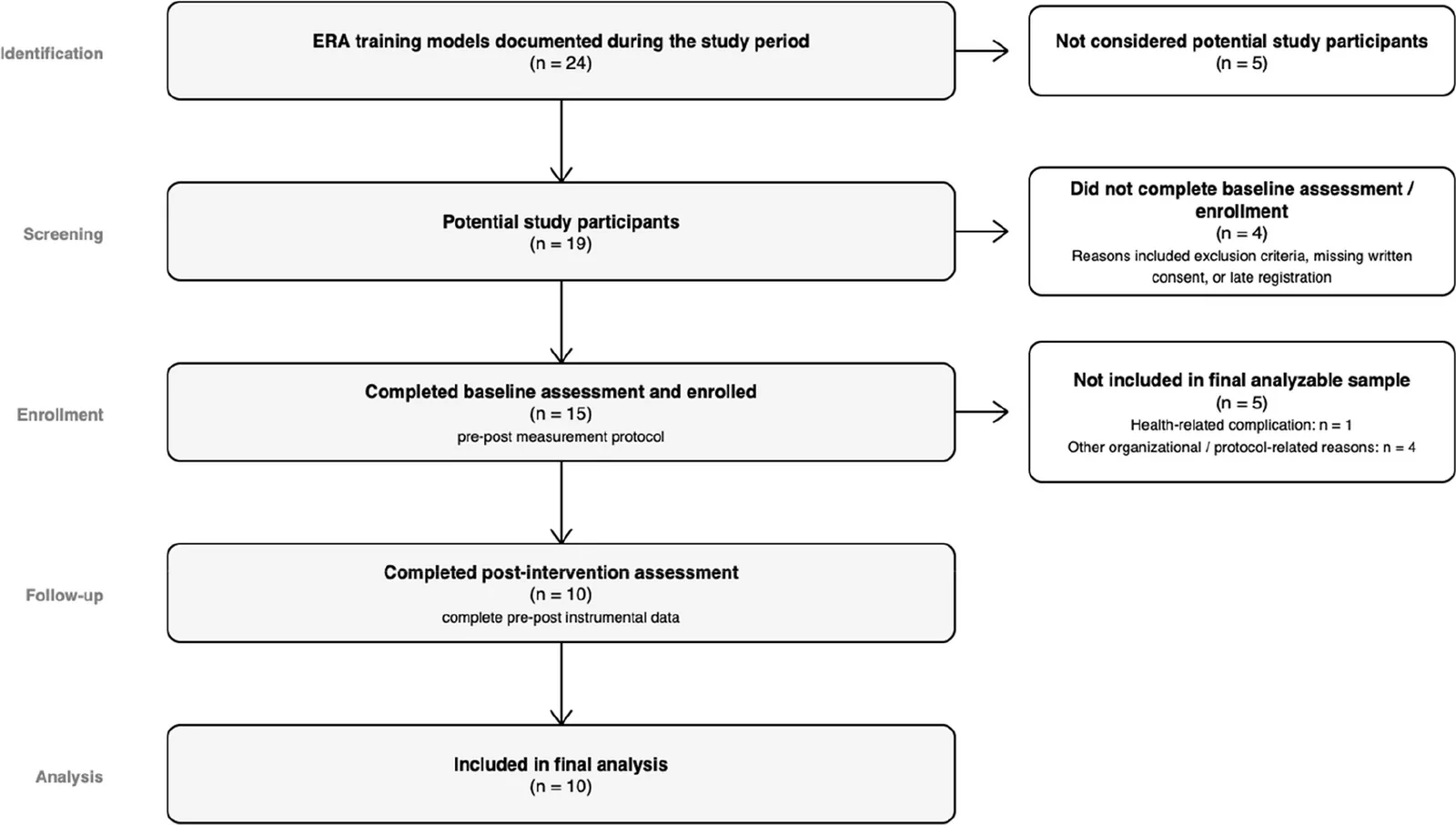
Participant flow diagram. CONSORT-style participant flow diagram for the single-arm pilot and feasibility study. Of 24 documented ERA training models, 19 were considered potential study participants; 15 completed baseline assessments, and 10 had complete baseline and post-intervention data and were included in the final analysis. *n* = number of individuals at each stage

During the study period, 24 ERA training models were documented, of whom 19 were considered potential study participants. Fifteen completed the baseline assessment and were enrolled in the pre–post protocol (78.9%). Ten participants completed both baseline and post-intervention assessments and were included in the final analysis, representing 66.7% of enrolled participants and 52.6% of potential participants. Losses before final analysis were due to one health-related discontinuation and study-specific or organizational reasons, including exclusion criteria, missing written consent, rebooking, late registration, or missed post-intervention assessment windows. Missing final-analysis data were primarily attributable to participant flow and scheduling rather than technical measurement failures. Figure 1 shows the participant flow.

### Baseline characteristics

Baseline characteristics are reported for the final analysis sample (*n* = 10). The mean age of participants with available data was 46.6 years (SD 12.3). Six participants were female, three were male, and sex was missing for one participant. Mean height was 173.4 cm (SD 13.1), mean weight was 70.6 kg (SD 12.3), and mean BMI was 23.5 kg/m^2^ (SD 3.5). Weight and BMI were summarized for nine participants because one participant reported body weight as a range rather than a single value. Age and sex were available for 9 participants because one participant’s first baseline questionnaire page was missing. Table 1 summarizes baseline characteristics, previous myofascial or manual treatment experience, and questionnaire-derived eligibility information.

**Table 1:** Baseline characteristics of the final analysis sample

Characteristic	Final analysis sample, *n* = 10
Age, years, mean ± SD	46.6 ± 12.3 (*n* = 9)
Age, years, median [range]	45 [32–64] (*n* = 9)
Sex, female, *n* (%)	6 (66.7%) (*n* = 9)
Sex, male, *n* (%)	3 (33.3%) (*n* = 9)
Height, cm, mean ± SD	173.4 ± 13.1
Height, cm, median [range]	172.0 [150–195]
Weight, kg, mean ± SD	70.6 ± 12.3^a^
Weight, kg, median [range]	70.5 [54–86]^a^
BMI, kg/m^2^, mean ± SD	23.5 ± 3.5^a^
BMI, kg/m^2^, median [range]	23.2 [19.1–30.1]^a^
Participation as ERA training model, *n* (%)	10 (100%)
Previous myofascial/manual treatment experience reported, *n* (%)	7 (70%)
No previous myofascial/manual treatment reported, *n* (%)	2 (20%)
Previous treatment unclear or missing, *n* (%)	1 (10%)
No exclusion-relevant medication reported, *n* (%)	9 (90%)
Medication entry unclear, *n* (%)	1 (10%)
No chronic/autoimmune disease reported, *n* (%)	9 (90%)
Chronic/autoimmune disease reported, *n* (%)	1 (10%)
No listed exclusion criterion reported, *n* (%)	10 (100%)
Relevant functional limitation noted in free text, *n* (%)	1 (10%)

*BMI *body mass index, *ERA* European Rolfing Association, *SD *standard deviation

^a^Weight and BMI were summarized for *n* = 9 because one participant reported body weight as a range (80–85 kg) rather than a single value

### Exploratory DIERS z-normalized postural imbalance index

The DIERS z-normalized postural imbalance index was summarized as an exploratory descriptive outcome. Because the index has not been biomechanically validated and used equal weighting of three DIERS parameters, it was not treated as a validated clinical endpoint or as evidence of intervention efficacy. The score changed from 2.46 (SD 2.83) at baseline to 2.23 (SD 2.62) after the RSI 10-series. The mean pre–post change was −0.23 points. These descriptive values did not indicate a clear group-level change in the combined postural imbalance score.

The three individual DIERS components that contributed to the index were also examined descriptively. Pelvic inclination decreased from 16.10° (SD 6.17) to 14.20° (SD 6.60), with a mean change of −1.90°. Trunk inclination decreased from 2.60° (SD 3.10) to 0.80° (SD 1.99), with a mean change of −1.80°. Trunk imbalance changed from −9.00 mm (SD 7.93) to −6.90 mm (SD 5.86), with a mean change of 2.10 mm. Because this was a small, uncontrolled pilot and feasibility study, interpret these descriptive instrumental findings only as information about outcome variability and measurement feasibility, not as evidence of intervention efficacy. The DIERS composite index should be interpreted with caution because it is unvalidated and based on equal weighting of heterogeneous postural parameters. Complete descriptive statistics for all instrumental outcomes are provided in Supplementary Table S2.

### Secondary and exploratory tissue and sensory outcomes

Pressure pain threshold showed divergent descriptive changes between the peripheral comparison site and the lower-back measurement site. At the forearm, pressure pain threshold decreased from 58.12 N (SD 19.14) at baseline to 49.70 N (SD 21.96) post-intervention, with a mean change of −8.41 N. At the lower back, pressure pain threshold increased from 71.62 N (SD 27.93) to 91.72 N (SD 56.94), with a mean change of 20.10 N. These exploratory results did not indicate a clear group-level change.

Local skin temperature at the lower back remained largely unchanged, with values of 35.28 °C (SD 1.20) at baseline and 35.52 °C (SD 1.00) post-intervention. The mean change was 0.24 °C.

MyotonPro-derived lower-back stiffness showed no clear group-level pre–post change, increasing slightly from 331.50 N/m (SD 45.85) to 336.20 N/m (SD 51.91), with a mean change of 4.70 N/m. MyotonPro frequency decreased from 16.65 Hz (SD 1.40) to 16.40 Hz (SD 1.92), with a mean change of −0.25 Hz.

IndentoPro-derived lower-back stiffness decreased from 1.44 N/mm (SD 0.44) to 1.38 N/mm (SD 0.23). The mean change was −0.07 N/mm. Overall, secondary and exploratory tissue, sensory, and temperature outcomes showed no clear group-level pre–post changes in this final analysis sample.

### Individual response patterns

Individual participant trajectories were inspected descriptively because group-level means may obscure divergent response patterns in small pilot samples. This inspection was performed across the main postural outcome and all secondary instrumental outcomes. The largest ranges of individual change were observed for stiffness-related outcomes and lower-back pressure pain threshold; therefore, these outcomes are reported as descriptive examples of interindividual variability rather than as separate inferential findings.

MyotonPro-derived lower-back stiffness showed changes ranging from −98 N/m to +84 N/m, with decreases in 4 participants and increases in 6 participants. IndentoPro-derived lower-back stiffness also showed bidirectional changes, ranging from −0.88 N/mm to +0.52 N/mm, with decreases in 5 participants and increases in 5 participants. Pressure pain threshold in the lower back showed changes ranging from −42.87 N to +168.05 N, with increases in 7 participants and decreases in 3.

These findings were not treated as separate inferential outcomes or graphically highlighted because the analyses were exploratory and not predefined for individual-response interpretation. The absence of clear group-level changes should therefore be interpreted alongside individual-level variability, while avoiding overinterpretation of selected individual responses.

### Patient-reported outcomes and adverse-event

At mid-series, categorical data on low-back-related activity restriction were available for all 10 participants. Nine participants reported 0 days of activity restriction due to lower-back problems during the preceding week, and one participant probably reported 1 day. At follow-up, clearly interpretable categorical data were available for 8 participants: six reported 0 days and two reported 1 day of activity restriction. Two follow-up entries could not be classified reliably from the scanned forms.

For low-back-related restriction of usual activities, the clearly marked responses most indicated less than half a day of restriction. No serious adverse event was documented in the available mid-series or follow-up questionnaire records. Patient-reported visual analog scale ratings for lower-back mobility and lower-back discomfort were not included in the quantitative analysis because the scanned line markings could not be transcribed with sufficient numerical precision. This represents an important and preventable limitation of the patient-reported outcome data-capture procedure. Therefore, only clearly identifiable categorical questionnaire items were summarized, and future studies should use digital VAS capture or higher-resolution source-data preservation to ensure quantitative analyzability.

## Discussion

This single-arm pilot and feasibility study evaluated whether a supervised RSI 10-series delivered in an educational training setting could be combined with a multimodal pre–post measurement protocol. Overall, the study showed that the intervention and several instrumental assessments could be completed in participants who remained in the study, but it also identified important feasibility challenges related to participant flow, scheduling, DIERS measurement standardization, provider-background documentation, and quantitative patient-reported outcome capture. The study was not designed or powered to evaluate RSI efficacy, and no conclusions about treatment effects can be drawn.

### Principal findings

The principal findings concern feasibility of study conduct and measurement implementation rather than intervention efficacy. Recruitment and baseline assessment were feasible within the educational training setting, but attrition and scheduling constraints reduced the final analyzable sample to 10 participants with complete pre–post instrumental data. Among participants who remained in the study, the RSI 10-series and post-treatment instrumental assessments could be completed.

Several measurement-related feasibility issues affected interpretability. These included DIERS standardization challenges, incomplete documentation of provider background, loss of quantitative visual analog scale data due to insufficient scan precision, and limited interpretability of two follow-up activity-restriction questionnaire entries. The DIERS-derived z-normalized postural imbalance index was treated as a strictly exploratory descriptive measure and should not be interpreted as a validated clinical endpoint or as evidence of RSI efficacy. Observed pre–post differences in instrumental outcomes should therefore be used only to describe feasibility, data quality, and outcome variability in this pilot sample.

### Relation to existing literature

The findings should be interpreted in the context of the current RSI literature, which has expanded in recent years but remains heterogeneous and insufficient for drawing conclusions about effectiveness. Previous studies have examined RSI or RSI-related interventions in selected domains and populations, including retrospective range-of-motion data after 10 RSI sessions and randomized investigations in specific clinical or functional contexts [10–12]. However, the present study was not designed as an effectiveness trial but as a prospective pilot and feasibility study focusing on the implementation of a broader technology-based measurement battery around a complete RSI 10-series. This focus is relevant because much of the existing RSI literature has relied on range-of-motion measures, subjective reports, or population-specific clinical outcomes. The present study adds to the literature by examining whether objective postural, sensory, physiological, and biomechanical measurements can be integrated into a full 10-session intervention delivered in an educational training context. The use of DIERS formetric 4D rasterstereography is of particular interest because objective postural assessment may provide complementary information to subjective reports and simple range-of-motion outcomes. Previous reliability research generally indicates good reliability for many DIERS spine-shape parameters, although reliability varies across individual parameters [18]. At the same time, this pilot also demonstrates that technology-based outcomes require rigorous procedural standardization, especially when repeated measurements are collected across several weeks in an on-site training environment rather than in a highly controlled laboratory setting.

### Methodological strengths and limitations

A strength of this study is its prospective pre–post design around the complete RSI 10-series rather than an isolated single-session or single-technique intervention. The intervention, therefore, reflected the traditional structure of RSI more closely than studies focusing only on short-term or local effects. Another strength is the use of a broad battery of instrumental measurements covering postural alignment, pressure pain sensitivity, local skin temperature, and biomechanical tissue properties. This approach provides practical information about the feasibility of collecting objective outcomes in future RSI research.

The educational training setting is also methodologically relevant. Treatments were delivered by Rolfers-in-training under senior supervision and were embedded in the standard structure of an Intensive Basic Rolfing® Training. This differs from routine clinical practice because the intervention was delivered within a structured educational context and was subject to training-related scheduling and organizational constraints. The findings should therefore be generalized primarily to comparable educational or trainee-delivered RSI settings and only cautiously to routine clinical practice. Several limitations should be considered. The final analyzable sample was small, which limits statistical power and precision of estimates. However, this limitation should be interpreted in light of the pilot and feasibility purposes of the study. The main value of the study lies not in estimating treatment effects with sufficient precision, but in identifying procedural challenges and informing the design of future research. Participant flow and scheduling within the educational training setting reduced the final analyzable sample and represent one of the most important feasibility findings. Reasons for missing final-analysis data included eligibility-related issues, missing written consent, rebooking of the RSI 10-series, late registration as a training model, health-related discontinuation, and non-completion of the post-measurements within the required assessment window. These issues indicate that future studies in comparable settings need careful recruitment tracking, scheduling coordination, retention procedures, and timely post-treatment assessment.

The standardization of DIERS measurements emerged as a major feasibility limitation. Practical challenges in the on-site setting included marker placement and adhesion, varying device-height settings, and uncertainty about how these factors affected repeated measurements. In some participants, additional DIERS recordings were obtained when the initial recording was considered insufficient for further processing. The available measurement files were later reviewed to identify comparable baseline and follow-up recordings for descriptive analysis, particularly regarding marker placement and device height settings. This file-selection process was not blinded to measurement time point and was not based on predefined exclusion or retention criteria. The exact number of unsuitable or excluded DIERS recordings was not documented as a separate feasibility variable. Intrarater or interrater reliability of DIERS measurements was not assessed in the current sample. These issues limit the interpretability of the preliminary DIERS findings and should be regarded as central feasibility findings rather than minor procedural details. The intervention itself also presents methodological challenges. The RSI 10-series follows a standardized session sequence, but the specific manual contacts, pressure, pacing, tissue targets, and movement or perception cues are adapted to the individual participant. This individualization is an inherent part of RSI and should not be removed through excessive manualization. However, for research purposes, a minimal level of session-level documentation may still be useful if it is compatible with the educational setting and the nature of the intervention. Such documentation should respect the individualized character of the work while recording enough information to describe treatment delivery, supervision, and any major deviations from the intended session goals.

Respiratory phase was not specified as a controlled variable during lumbar indentometry. Although respiratory-related trunk movement could represent a potential source of variability, respiratory-phase control is not routinely reported in many indentometry protocols. Future studies may nevertheless consider documenting breathing instructions or respiratory phase to further improve measurement standardization.

The available dataset also did not systematically document trainees’ prior clinical, manual therapy, or bodywork experience. This is an important provider-level feasibility limitation because prior experience may influence intervention delivery, including manual contact, pacing, pressure, tissue selection, and movement or perception cues. The absence of these data means that provider background could not be described quantitatively or taken into account when interpreting intervention delivery in this trainee-based setting. Future studies should therefore document trainee background, prior clinical or manual therapy experience, supervision context, and, where feasible, participant-to-trainee assignment while still respecting the individualized nature of RSI. Finally, multiple exploratory outcomes were examined descriptively. This aligns with the purpose of the pilot study, but it means that observed changes should not be interpreted as confirmatory. In particular, the descriptive decrease in trunk inclination should be reported only as an observed pre–post difference and should not be interpreted as evidence of RSI-related postural improvement or intervention effectiveness. In addition, the loss of quantitative VAS data due to scanned line markings that could not be transcribed with sufficient precision represents a preventable data-management limitation. Future studies should avoid unnumbered scanned VAS line markings and should instead use digital VAS capture or a numbered 0–10 or 1–10 response format to ensure quantitative analyzability of patient-reported outcomes.

### Methodological implications

The present findings have methodological rather than clinical implications. They indicate which elements of a multimodal measurement protocol may be feasible in an educational RSI training setting and which elements require optimization before evaluation in an adequately powered controlled trial. The lower-back tissue-property measures should be understood as exploratory local measurements in an anatomically relevant and practically accessible region related to the thoracolumbar fascia, not as proxies for whole-body RSI effects or clinical mechanisms. Testing feasibility in non-clinical training models may nevertheless be a pragmatic first step before applying a complex and potentially burdensome measurement protocol in future clinical or controlled research. The study also has educational relevance because it examines whether research assessments can be integrated into a supervised trainee-delivered 10-series within a structured training environment.

### Future research directions

The findings from this pilot study provide a basis for refining the protocol before progression to a controlled trial. Several elements appeared feasible among participants who remained in the study, including completion of the RSI 10-series and attendance at post-treatment instrumental assessments. However, progression to a larger study would require optimizing participant flow, scheduling within the educational training setting, DIERS measurement standardization, provider background documentation, quantitative VAS capture, and handling of unclear questionnaire entries.

The target population should be defined a priori in subsequent work. Because the present study was conducted in non-clinical training models, its findings are most directly applicable to the planning of feasibility research in comparable educational or non-clinical RSI settings. Transfer to clinical populations, such as people with persistent non-specific low-back pain or other musculoskeletal complaints, would require separate feasibility testing, population-specific eligibility criteria, appropriate outcome selection, and safety monitoring.

Protocol refinement should focus first on recruitment and retention procedures, clearly defined post-treatment assessment windows, and prospective documentation of the training and supervision context. Intervention-delivery documentation should include trainee background, supervision arrangements, and key session-level information where this can be captured without compromising the individualized nature of RSI. Rather than attempting to fully manualize the intervention in a way that would alter its character, future research should describe the standardized 10-series framework clearly and document treatment delivery at a level compatible with educational or clinical practice.

DIERS procedures require particular attention before use in a larger study. Device setup, participant positioning, marker or landmarking procedures, and measurement conditions should be standardized and documented prospectively. Criteria for handling repeated, technically insufficient, or deviating recordings should be defined before data collection begins, and blinded or independent review of measurement files should be considered where feasible. Because practical deviations may still occur in educational or real-world settings, protocols should also specify how such deviations will be documented, evaluated, and incorporated into the analysis.

The current DIERS composite score should not be carried forward as a primary or preferred postural outcome. If postural outcomes are included, emphasis should be placed on established individual DIERS parameters or other individual kinematic and postural variables that are selected a priori, biomechanically justified, and interpreted descriptively or within an adequately powered controlled design. Any composite postural index would require independent methodological development and validation before being used as a primary endpoint or as a summary measure of postural change.

Patient-reported outcomes, participant burden, and adverse-event capture should be integrated prospectively into the study workflow and monitored for completeness. In particular, unnumbered scanned VAS line markings should be avoided; digital VAS capture or numbered response formats would improve quantitative analyzability. Reliable capture of participant experiences and potential adverse events is integral to feasibility in manual therapy research and should be treated as a core procedural outcome rather than an optional supplement to instrumental measurements.

Once feasibility procedures, outcome selection, measurement standardization, intervention-delivery documentation, and patient-reported data capture have been optimized, the protocol could be considered for evaluation in an adequately powered controlled study.

## Conclusion

This single-arm pilot and feasibility study showed that recruitment, completion of the RSI 10-series, post-treatment assessment attendance, and collection of several instrumental outcomes were feasible among participants who remained in the study. At the same time, the study identified important feasibility limitations, including participant flow and scheduling challenges, incomplete capture of patient-reported and adverse-event data, and procedural standardization issues, particularly related to DIERS measurements. Because the study was conducted in a small, non-clinical sample without a control group, the findings do not demonstrate the effectiveness or efficacy of RSI. Observed pre–post changes should therefore be interpreted only as preliminary descriptive feasibility data. Future controlled studies should refine recruitment and retention procedures, improve prospective documentation of intervention delivery and measurement standardization, and select outcomes a priori before evaluating RSI effects in an adequately powered clinical or non-clinical population.

## Supplementary Information

Supplementary Material 1.

## Data Availability

The datasets generated and/or analyzed during the current study are available from the corresponding author upon reasonable request.
