# Supplementary material for: Feasibility of measuring lumbar, postural, and biomechanical outcomes before and after a Rolfing® Structural Integration 10-series delivered by trainees: a single-arm pilot study
Source: Pilot Feasibility Stud. 2026 Nov 10;12:160. doi: 10.1186/s40814-026-01954-z (PMC13749995; doi:10.1186/s40814-026-01954-z)
Supplement: Supplementary file 1 [file 40814_2026_1954_MOESM1_ESM.docx]

**Supplementary Material S1**

**Patient-Reported Questionnaires and Treatment-Related Experiences**

This supplementary material summarizes the German-language patient-reported questionnaires used in the Rolfing® Structural Integration pilot and feasibility study. The content is presented in English to support the manuscript's reproducibility while preserving the structure of the original questionnaires.

**Table S1. Overview of questionnaire timing and purpose**

| **Time point** | **Questionnaire focus** | **Administration** |
| --- | --- | --- |
| Baseline | Eligibility-related information, participant characteristics, previous myofascial treatment experience, recent physical activity, lower-back mobility, pain, and activity restriction. | Completed before the start of the Rolfing® 10-series. Participants were instructed to answer the questionnaire undisturbed and without influence from others. |
| Mid-series | Lower-back mobility, pain, activity restriction, and treatment-related positive or negative experiences, including unwanted medical events. | Administered after the fifth Rolfing® session and returned before or at the sixth session. |
| Follow-up | Repeated patient-reported items on lower-back mobility, pain, activity restriction, and restricted usual activities due to lower-back pain. | Completed after completion of the Rolfing® 10-series in conjunction with the post-treatment assessment. |

**Table S2. Baseline questionnaire content**

| **Domain** | **Items / response content** |
| --- | --- |
| Administrative information | Participant identifier; sex; date of birth; date and signature. |
| Training-model status | Confirmation of participation as a model in the Rolfing® Training at the European Rolfing® Association Training Center. |
| Potential participation-relevant impairments | Hearing, vision, speech, or mobility impairments that could affect study participation. |
| Health conditions and contraindication-related items | Pregnancy; eczema; skin inflammation; psoriasis; burns; acute trauma; unhealed fractures; open wounds; osteoporosis; discectomy; implanted screws, plates, wires, or medical implants such as a pacemaker; hypercoagulability; deep venous thrombosis; fibromyalgia; insufficiently controlled psychiatric illness. |
| Medication and substance-related items | Anticoagulant medication; immunosuppressive medication; chronic steroid medication; daily analgesic use; alcohol or substance misuse. |
| Chronic diseases | Type I diabetes; Crohn’s disease; lupus; inflammatory bowel disease; autoimmune disease; cancer; other chronic diseases. |
| Anthropometrics | Body height in cm and body weight in kg. |
| Previous myofascial treatment experience | Previous experience with myofascial treatment, such as Rolfing®, foam rolling, or similar approaches. |
| Recent physical activity | Type and extent of physical activity during the previous three days. |
| Subjective lower-back mobility | Visual analogue-type rating of current lower-back mobility, anchored from very good to very poor. |
| Current lower-back pain / well-being | Visual analogue-type rating of current lower-back status, anchored from no pain to maximum pain. |
| Activity restriction due to lower-back problems | Number of days during the previous week on which physical activity was restricted because of lower-back problems, from 0 to 7 days. |
| Restricted usual activities due to lower-back pain | Number of half-days during the previous week spent in bed, on sick leave, or with restricted usual activities because of lower-back pain, from no half-day to 14 half-days. |

**Table S3. Mid-series questionnaire content**

| **Domain** | **Items / response content** |
| --- | --- |
| Subjective lower-back mobility | Visual analogue-type rating of current lower-back mobility, anchored from very good to very poor. |
| Current lower-back pain / well-being | Visual analogue-type rating of current lower-back status, anchored from no pain to maximum pain. |
| Activity restriction due to lower-back problems | Number of days during the previous week on which physical activity was restricted because of lower-back problems, from 0 to 7 days. |
| Restricted usual activities due to lower-back pain | Number of half-days during the previous week spent in bed, on sick leave, or with restricted usual activities because of lower-back pain, from no half-day to 14 half-days. |
| Treatment-related experiences | Participants marked only experiences that they definitely had during or after the previous treatment. For each endorsed experience, duration during treatment and duration after treatment were recorded using predefined categories. Free-text fields allowed further description. |

**Table S4. Duration categories for treatment-related experiences**

| **Occurrence period** | **Duration categories** |
| --- | --- |
| During treatment | A few minutes; more than a few minutes; most of the treatment. |
| After treatment | A few minutes; more than a few minutes up to one hour; more than one hour; approximately one day; several days; one week or longer. |

**Table S5. Treatment-related experiences assessed in the mid-series questionnaire**

| **Category** | **Experiences assessed** |
| --- | --- |
| Sensory experiences | Numbness; tingling; warmth/heat; cold. |
| Autonomic or respiratory experiences | Sweating; rapid breathing; very slow breathing or brief pauses in breathing. |
| Vestibular or gastrointestinal experiences | Dizziness or vertigo; nausea. |
| Emotional or memory-related experiences | Perception of an emotion; memory of the past. |
| Motor or body-state experiences | Trembling or shaking; feeling that the body was pulsating; feeling of great lightness or weightlessness; feeling heavier. |
| Relaxation and pleasant bodily experiences | Deep relaxation; pleasant warmth. |
| Coordination and stability | Feeling of better coordination while walking; feeling of worse coordination while walking; feeling more secure on the feet; feeling less secure on the feet. |
| Breathing and movement | Easier breathing; more difficult breathing; easier movement; more difficult movement. |
| Body perception | Feeling of having left the body; feeling more present in the body. |
| Pain-related experiences | Stabbing pain; burning pain; dull pain. |
| Other experiences | Free-text description of any other positive or negative experience, including onset and duration. |

**Table S6. Follow-up questionnaire content**

| **Domain** | **Items / response content** |
| --- | --- |
| Administrative information | Participant identifier; date; signature. |
| Subjective lower-back mobility | Visual analogue-type rating of current lower-back mobility, anchored from very good to very poor. |
| Current lower-back pain / well-being | Visual analogue-type rating of current lower-back status, anchored from no pain to maximum pain. |
| Activity restriction due to lower-back problems | Number of days during the previous week on which physical activity was restricted because of lower-back problems, from 0 to 7 days. |
| Restricted usual activities due to lower-back pain | Number of half-days during the previous week spent in bed, on sick leave, or with restricted usual activities because of lower-back pain, from no half-day to 14 half-days. |
